# Bilateral Subtrochanteric Complete Atypical Femoral Fracture in a Patient With Rheumatoid Arthritis: A Case Report

**DOI:** 10.7759/cureus.30692

**Published:** 2022-10-26

**Authors:** Youngho Cho, Jae Uk Shin

**Affiliations:** 1 Orthopedics and Trauma, Daegu Fatima Hospital, Daegu, KOR

**Keywords:** osteoporosis, rheumatoid arthritis, atypical femoral fracture, subtrochanteric fracture, bilateral fractures

## Abstract

Antiresorptive drugs such as bisphosphonates (BPs) or denosumab, used for the treatment of osteoporosis over the past decades, have improved bone mineral density and reduced the incidence of fractures. However, there are increasing evidence that atypical femoral fractures (AFFs) are related to long-term use of these medications.

We had experienced bilateral simultaneous subtrochanteric complete AFFs in having rheumatoid arthritis (RA) for 15 years. She just had been taking risedronate for three months prior to this event. Fractures were treated with long cephalomedullary nails. We could get a bone union for the right side at 15 months after index surgery. However, two more surgeries were needed to get bone union for the left side. This study aimed to share our treatment strategy and review of the literature on the correlation between RA and AFFs.

## Introduction

Bisphosphonates (BPs) have been widely used for prevention of osteoporosis and related fractures for the last few decades. However, long-term usage makes adverse effects such as atypical femoral fractures (AFFs) [1-3]. A denosumab which is also a potent antiresorptive agent is also related to AFFs though the incidence is lower than that of BP [4].

Rheumatoid arthritis (RA) patients have several risk factors for osteoporosis like older age, lower body mass index, longer disease duration, and long-term exposure to glucocorticoids [5]. Eventually, the risk of osteoporotic fractures increases [6]. Therefore, there is a tendency to use antiosteoporosis drugs from a young age compared to general population, and the risk of side effects from long-term usage increases.

There were previous reports about bilateral complete atypical femoral fractures in association with BPs or denosumab [7-12]. We could not confirm prior reports of the case report of bilateral subtrochanteric complete AFFs in a RA patient with short-term BPs exposure. The purpose of our report is to share our treatment strategy and review of the literature on the correlation between RA and AFFs.

## Case presentation

A 66-year-old female patient presented to the emergency department with bilateral thigh pain after tripping over a threshold. Shortening and deformities were observed in both thighs and the patient complained of inability to walk. She had diffuse pain in both thighs for several months. However, she did not evaluate the pain but took a nonsteroidal anti-inflammatory drug under the assumption of lumbar spinal stenosis.

**Figure 1:**
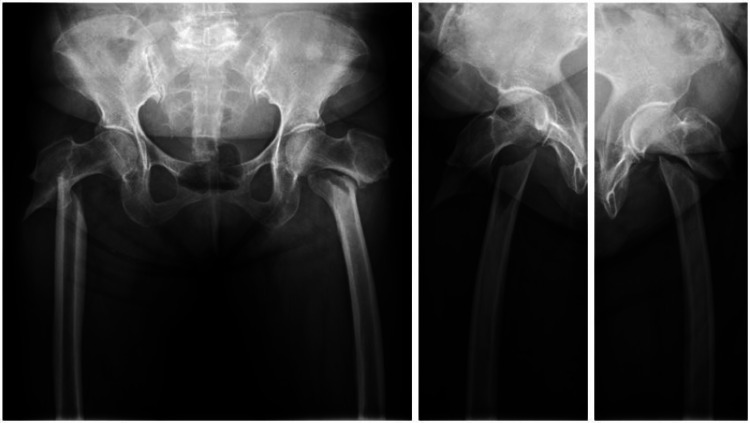
Anteroposterior and lateral radiographs of hip joint of a 66-year-old female with bilateral subtrochanteric complete atypical femoral fracture.

Radiographic study showed bilateral subtrochanteric fractures with complete displacement (Figure 1). On the right side, the fracture line was short transverse at the lateral cortex and extended obliquely to the proximal medial diaphysis. On the left side, the fracture line was short oblique with lateral cortical thickening at the level of lesser trochanter.

She had a seropositive RA and had been taking the medicine including methotrexate (MTX) for 15 years. At the time of injury, she had been taking methotrexate 10 mg once a week, triamcinolone 4 mg once a day, thiamine hydrochloride 10 mg once a day, celecoxib 200 mg once a day, gabapentin 300 mg once a day, and itopride hydrochloride 50 mg once a day. She couldn't remember how long each drug was taken. However, she said she had been taking osteoporosis medication (risedronate 35 mg) for the past three months. She underwent total knee arthroplasty on both sides two years ago and was able to do outdoor activities without any aid.

**Figure 2:**
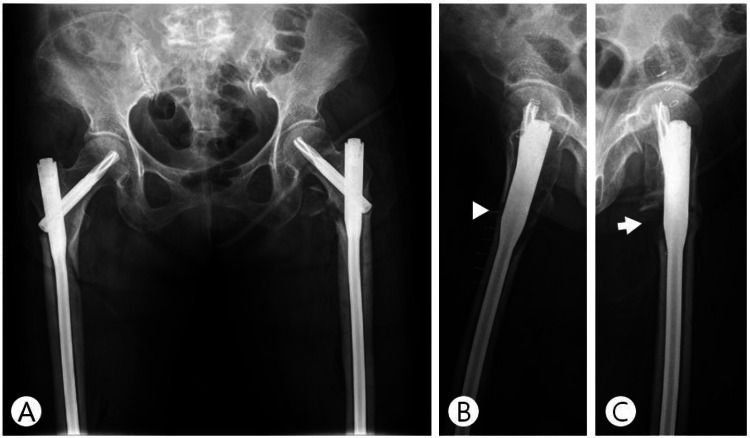
Postoperative radiographs of both femur subtrochanteric atypical fractures treated with long proximal femoral nail antirotation II. The images show (A) anteroposterior radiograph of both hip joints, (B) right lateral radiograph shows gap between fracture fragments (arrowhead), and (C) left lateral radiograph shows bone defect due to displaced lesser trochanteric fracture fragment (arrow).

In the preoperative dual-energy x-ray absorptiometry, the T-score was -1.3 in the lumbar spine. The serum 25-OH vitamin D was 7.9 ng/mL (range: 30.0-100.0 ng/mL). The bone formation marker osteocalcin and bone resorption marker C-telopeptide of type I collagen were measured to be 7.8 ng/mL (range: 15-46 ng/mL) and 0.348 ng/mL (0.177-1.015 ng/mL), respectively. Both femurs were treated with internal fixation using long proximal femoral nail antirotation II (PFNA-II; Oberdorf, Switzerland: Synthes GmbH). We obtained satisfactory reduction for the right femur. The left femur was reduced about 5° of varus compared to the right, and an avulsion fracture occurred in the lesser trochanter (Figures 2A-2C). All medications were maintained except risedronate. We gave oral calcium and vitamin D supplements to the patient.

**Figure 3:**
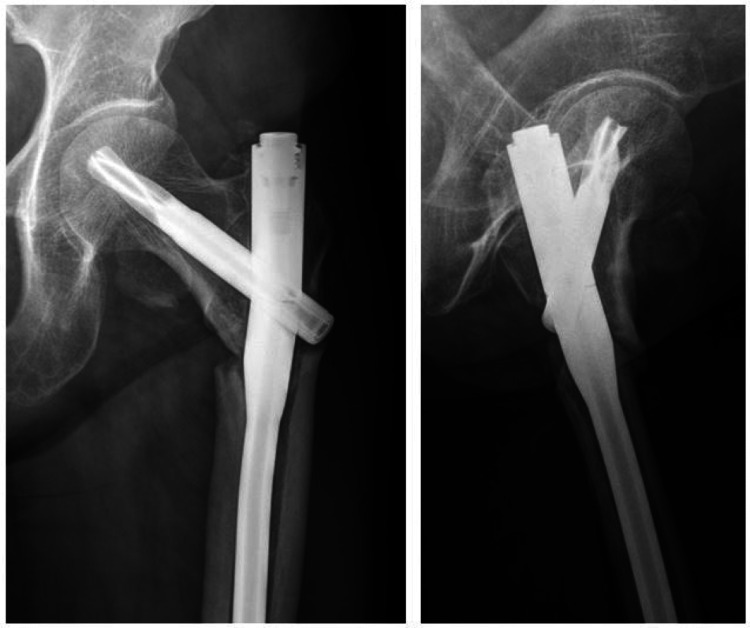
Anteroposterior and lateral radiographs of the left femur obtained 15 months after index surgery shows nail breakage which indicates nonunion.

**Figure 4:**
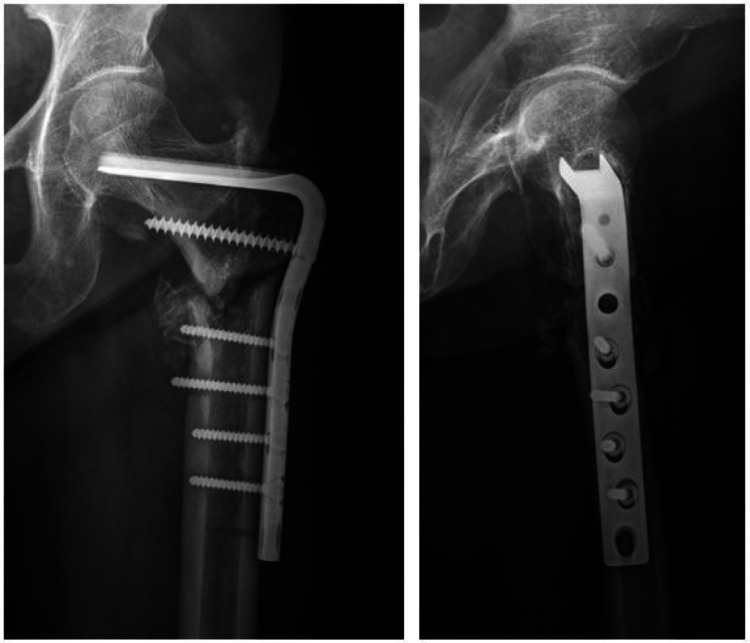
Revision surgery of left femur after fixation with condylar blade plate and autogenous cancellous bone graft shows slight residual varus alignment.

**Figure 5:**
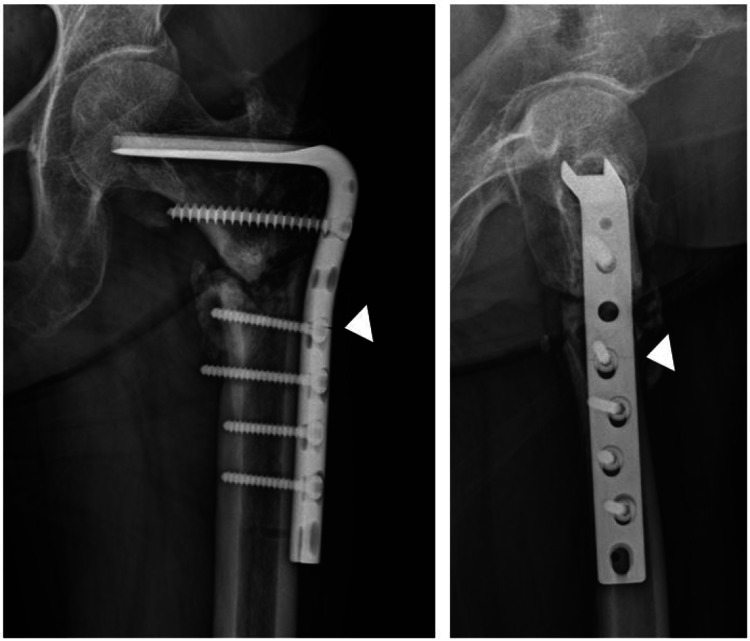
Simple radiographs 13 months after revision surgery shows breakage of plate (arrowhead).

**Figure 6:**
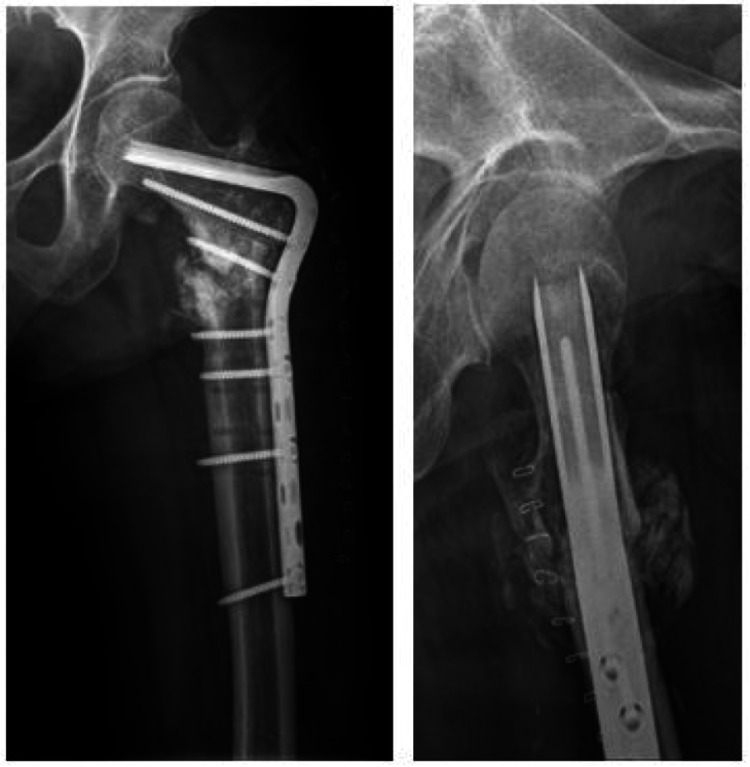
Radiographs after re-revision surgery shows corrected varus alignment and abundant bone chips which was made by decortication and autogenous cancellous bone graft.

**Figure 7:**
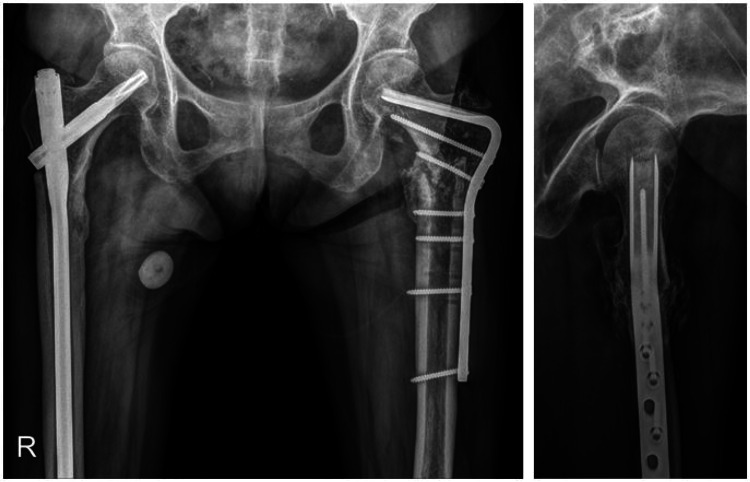
Simple radiographs seven months after re-revision surgery shows connection of fracture with external callus.

At the 15-month follow-up, the right side showed bridging callus formation and bony union was achieved. However, she was suffering from pain on the left side and radiographs showed breakage of intramedullary nail at the junction of nail and blade (Figure 3). Revision surgery was carried out as follows: removal of previous implant, internal fixation with angled blade plate and autogenous cancellous bone graft (Figure 4). A new pain developed 13 months after revision surgery. Breakage of the plate was observed on radiographs (Figure 5). Third operation was done. The fracture was exposed by Judet osteoperiosteal decortication technique. We corrected varus alignment and the fracture was refixed with the longer condylar blade plate. We also added autogenous cancellous bone graft (Figure 6). Bone union was achieved seven months after the third operation (Figure 7).

## Discussion

In 2005, the earliest report of a severely suppressed bone turnover and nontraumatic femoral fracture in a patient taking bisphosphonates was published [13]. Our patient had complete fractures of both femurs in subtrochanteric area simultaneously and all major diagnostic criteria of AFFs including minor trauma, subtrochanteric location of fracture, transverse or short oblique fracture line, no comminution, and medial spike lateral cortical thickening. Although our patient had both thigh pain before fractures, she was given only analgesic and antiinflammatory drugs under the assumption of lumbar spinal stenosis by the clinician. If there is pain in the thigh or groin in elderly patients taking BPs or denosumab regardless of the duration, physicians need to determine whether they have any clues of AFFs or not on radiographs.

According to the American Society for Bone and Mineral Research (ASBMR) report, RA is an associated medical condition mentioned in the minor features of AFFs [1]. Patients with RA are at increased risk of osteoporotic fractures. This increased risk is attributable to a combination of disease activity and use of oral glucocorticoids [6]. Also, RA patients who are receiving long-term (three to five years) BP treatment are at higher risk of AFF compared with matched control patients with RA [14]. In other words, RA patients are at risk of both osteoporotic fractures and AFFs. Sato et al. published a study on the correlation between prednisolone and AFFs [15]. They said that higher-dose prednisolone because of a comorbid disease requiring glucocorticoid treatment other than RA or refractory RA and methotrexate (MTX) were risk factors for localized periosteal thickening (associated with incomplete AFFs).

Our patient just took BP for three months. Therefore, the relationship between this event and BP is thought to be low. She took a glucocorticoid at the time of injury. However, we couldn’t find out exactly how much she had taken it in the past. So, we cautiously guess that RA itself or other medications they take may be related to AFF. This is an area that needs further research.

Intramedullary nail fixation is the widely accepted standard treatment for AFFs [16]. We also used blade-type cephallomedullary nails for both fractures. She could get a bone union on the right side after index surgery. However, two more surgeries were needed for left subtrochanteric fractures. We thought a slight varus reduction and bone defect made during the surgery were the main cause of nonunion and metal failure. Cho et al. reported high occurrence rates of nonunion and delayed union in patients with subtrochanteric AFFs. They said that a varus deformity greater than 4.4° in coronal plane or a sagittal angulation greater than 5.5° was associated with nonunion or delayed union [17]. Although a revision surgery was performed after 15 months after primary surgery, it also failed because a varus alignment wasn’t corrected. In the third operation, a varus deformity correction and osteoperiosteal decortication technique were added. Finally, the fracture healed seven months after the operation. Osterperiosteal decortication is effective for the treatment of atrophic or oligotrophic nonunion of the long bone diaphysis in the aspect of biology [18].

## Conclusions

We report a rare case of bilateral subtrochanteric complete atypical femoral fractures in a patient with RA. Long-term use of RA medication itself may be associated with AFFs, especially prednisolone and MTX. Even in RA patients who have taken BP for a short period of time, care should be taken for the risk of AFFs.
